# Limitations of Coronary Computed Tomography Angiography in Predicting Acute Coronary Syndrome in a Low to Intermediate-risk Patient with Chest Pain

**DOI:** 10.7759/cureus.2649

**Published:** 2018-05-18

**Authors:** Ahmed Zaghloul, Corina Iorgoveanu, Kathir Balakumaran, Dinu V Balanescu, Teodora Donisan

**Affiliations:** 1 Internal Medicine, University of Connecticut Health Center, Farmington, USA; 2 Cardiology, University of Connecticut Health Center, Farmington, USA; 3 Internal Medicine, Santa Maria Clinical Hospital, Santa Maria, USA; 4 Cardiology, Elias Emergency University Hospital

**Keywords:** acute coronary syndrome, coronary computed tomography angiography, coronary artery disease, coronary calcium scoring, major adverse cardiac events

## Abstract

The optimal management of patients with chest pain relies on the prognostic information provided by noninvasive cardiovascular testing. Coronary computed tomography angiography (CCTA) is an increasingly utilized, highly accurate noninvasive test for diagnosing coronary artery disease. We illustrate an exceptional limitation of the prognostic information provided by CCTA. A 46-year-old female presented with chest pain suggestive of angina. Noninvasive testing for ischemia was negative, with CCTA showing mild stenosis of the proximal left anterior descending (LAD) artery. An electrocardiogram after two weeks demonstrated ST elevation in leads V1-V2 and aVR, with ST depression in the lateral leads. Cardiac catheterization revealed a significant proximal LAD lesion requiring percutaneous coronary intervention. An anatomic assessment of coronary arteries should be considered in cases of strong clinical suspicion for cardiac ischemia and initial nondiagnostic findings. Further studies are needed to improve the accuracy and the negative predictive value of CCTA in intermediate-risk individuals.

## Introduction

Coronary artery disease (CAD) is the leading cause of mortality and morbidity in the modern world. The optimal management of patients with chest pain relies on the prognostic information provided by noninvasive cardiovascular testing [1], in the form of coronary computed tomography angiography (CCTA), as well as invasive coronary angiography. CCTA is an increasingly utilized, highly accurate, noninvasive test for diagnosing CAD. CCTA also provides prognostic information by evaluating the presence, extent, and location of both obstructive and non-obstructive coronary atherosclerosis. Our case presents an exceptional limitation of the prognostic information provided by CCTA and its ability to predict future plaque instability and, thereby, to prevent future major adverse cardiac events (MACE).

## Case presentation

**Figure 1:**
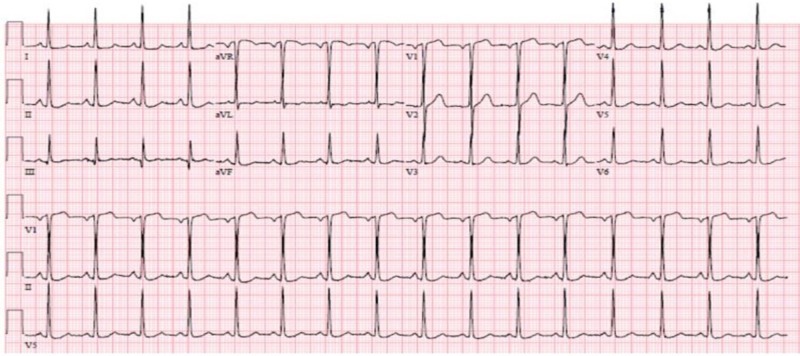
Electrocardiogram at the first presentation. Normal sinus rhythm without significant ST or T changes.

**Figure 2:**
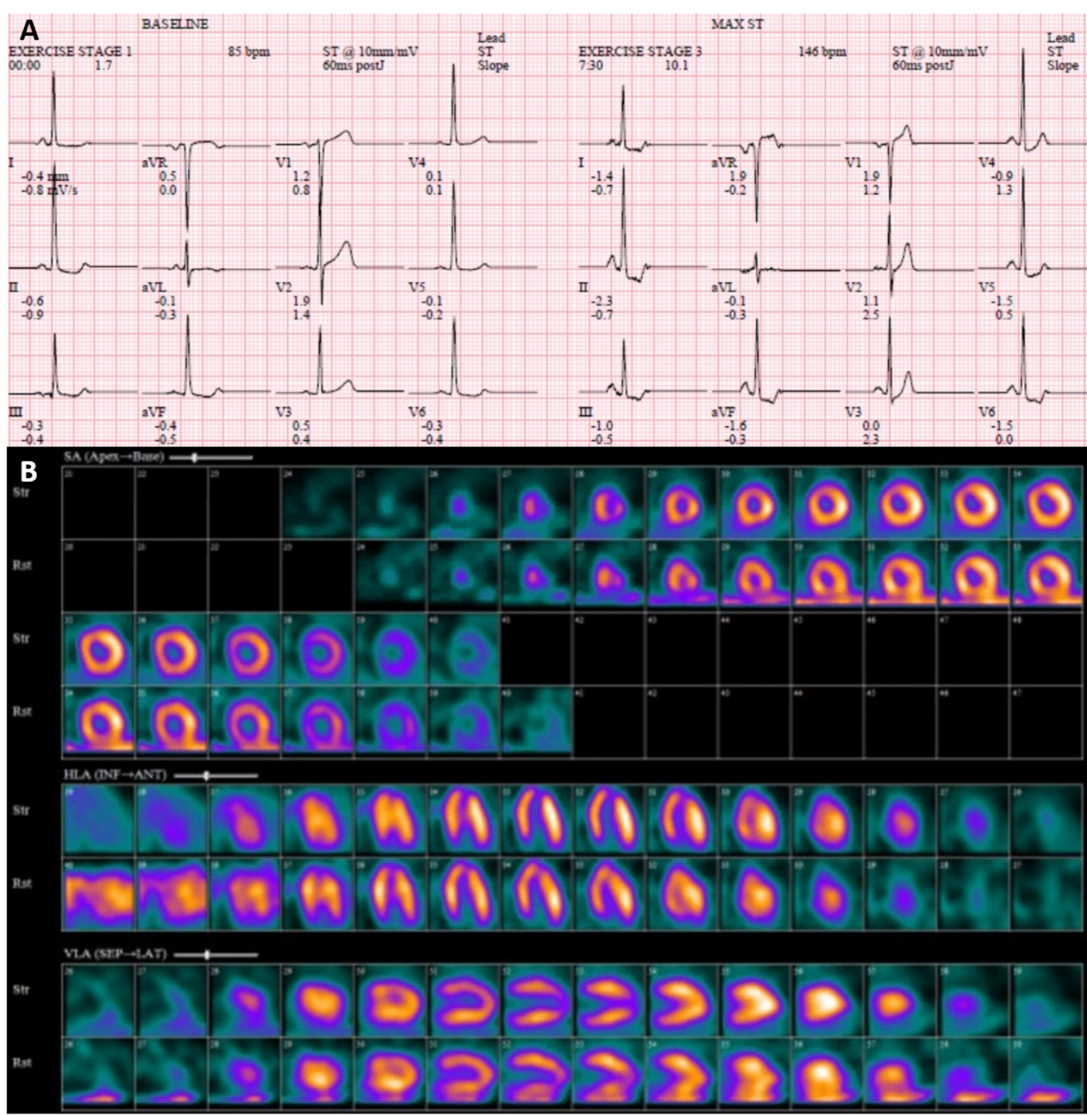
Exercise stress test. Normal electrocardiogram (A) and nuclear stress test (B).

**Figure 3:**
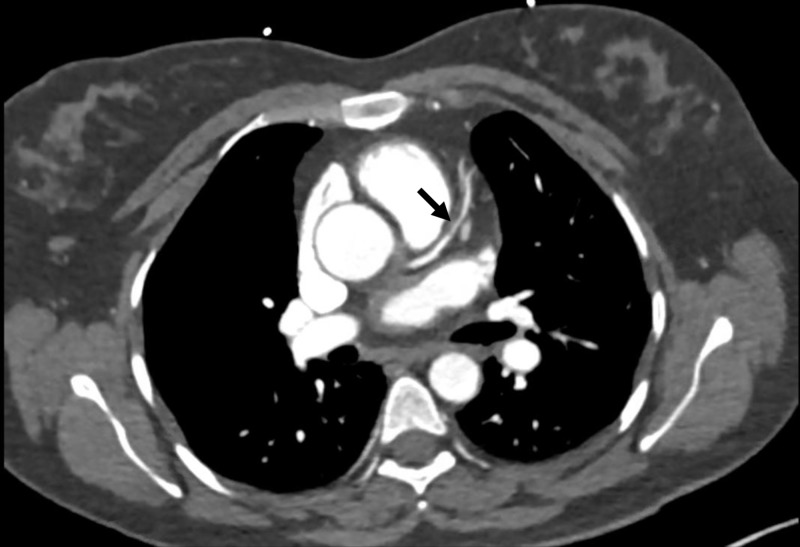
Coronary computed tomography angiography. Mild stenosis of the left anterior descending artery (arrow).

**Figure 4:**
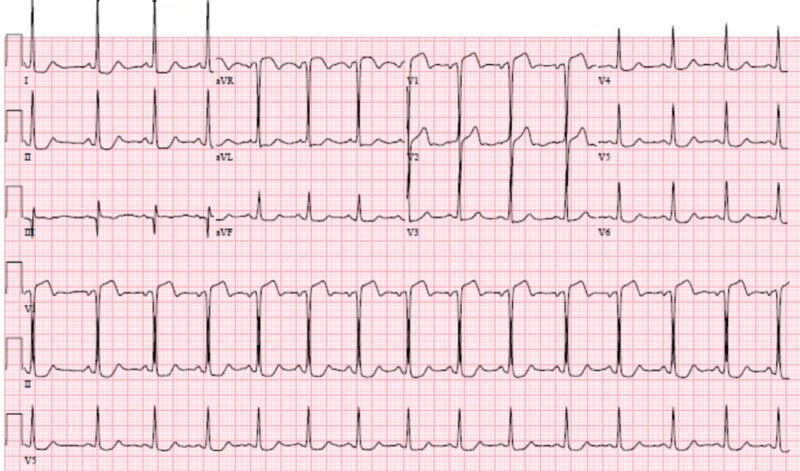
Electrocardiogram after two weeks. ST elevation in leads V1-V2 and aVR, with ST depression in the lateral leads.

**Figure 5:**
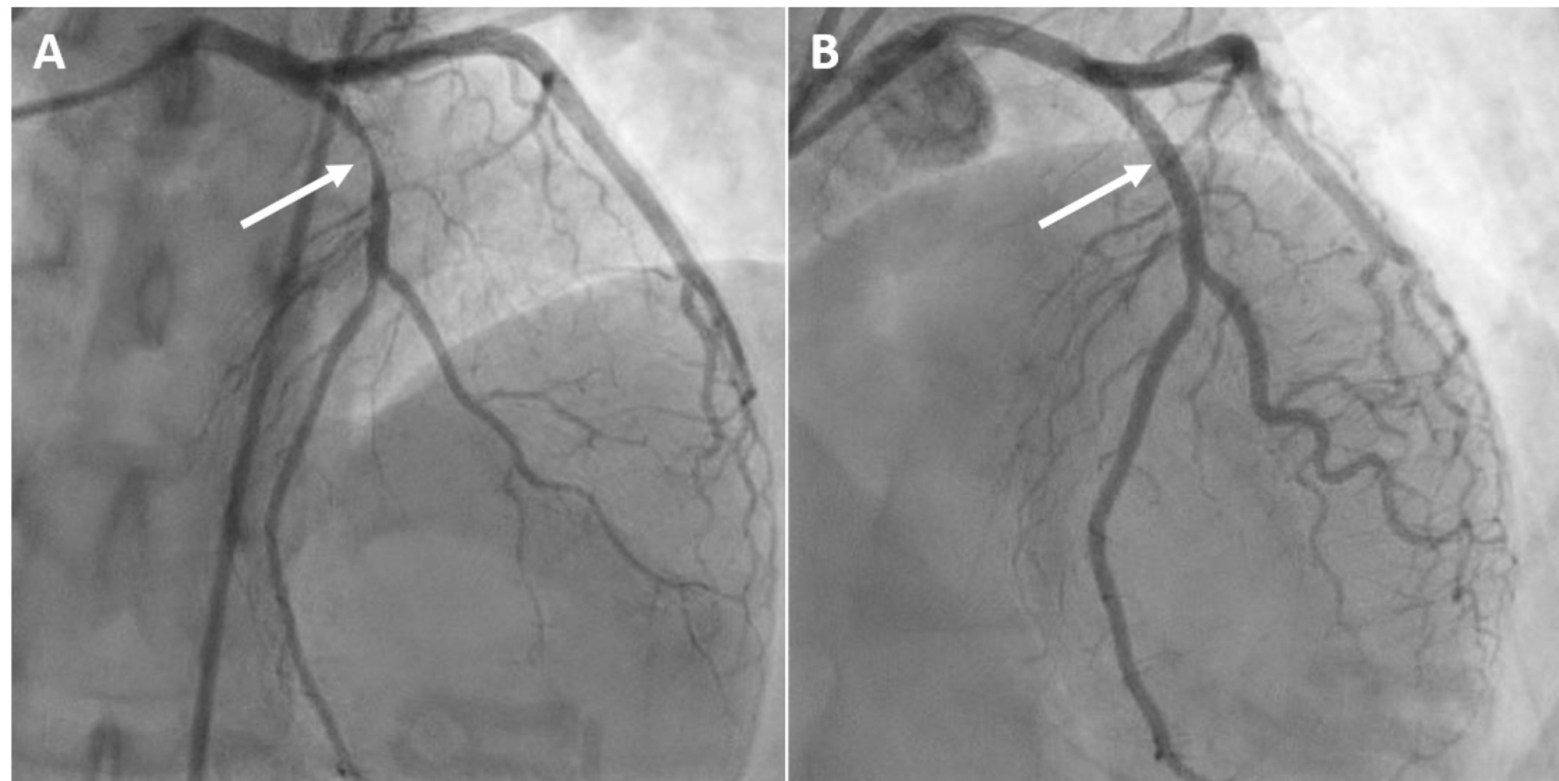
Coronary angiography with percutaneous coronary intervention. A significant proximal left anterior descending artery lesion (A, arrow) with the successful placement of a drug-eluting stent (B, arrow).

A 46-year-old female with a past medical history of essential hypertension and dyslipidemia, treated with amlodipine, hydrochlorothiazide, labetalol, and atorvastatin, presented to the emergency department (ED) with chest pain. Family history was non-significant for CAD or diabetes, and social history was remarkable for active tobacco smoking. The patient had been having intermittent substernal chest pain for two weeks. On the day of the presentation, she described an episode of acute onset, sub-sternal chest pain lasting for approximately 20 minutes while at her desk job. The pain had subsided gradually over the next 20-30 minutes, was not associated with any dyspnea, diaphoresis, nausea, vomiting, upper extremity or neck discomfort, or a headache. The patient drove herself to the hospital and while in the waiting room, she had a return of chest pressure that was similar to the earlier episode. Her chest pain improved greatly with nitroglycerin. An electrocardiogram (ECG) showed a normal sinus rhythm with left ventricular hypertrophy, with no significant ST or T wave changes (Figure 1). Cardiac biomarkers showed normal creatine kinase-muscle/brain (CK-MB) with mild troponin elevation (0.14 ng/ml), which subsequently normalized. Other labs were normal. Transthoracic echocardiography demonstrated a normal left ventricular size and function, with a normal ejection fraction (55%-65%) and wall motion. Nuclear exercise stress testing did not demonstrate ischemia (Figure 2). Because of high clinical suspicion, a CCTA was performed even though the stress test was negative; it did not reveal occlusive CAD, showing only a mild stenosis of the proximal left anterior descending (LAD) artery (Figure 3). She was discharged on aspirin and pantoprazole for suspected gastroesophageal reflux disease. After two weeks, the patient returned to the ED complaining of a similar chest pain, occurring at rest, with radiation to the neck, and partial response to nitroglycerin. The ECG demonstrated ST elevation in leads V1-V2 and aVR, with ST depression in the lateral leads (Figure 4). The patient was emergently taken to the cardiac catheterization lab and was found to have a significant proximal LAD lesion, requiring a percutaneous coronary intervention (PCI) with the placement of a drug-eluting stent (Figure 5). Post PCI, the chest pain and ECG changes resolved. Troponin levels peaked at 3.1 ng/ml. The patient had an uneventful course in the hospital. She was discharged after two days on dual antiplatelet treatment, high-dose statin, and a beta blocker, with a close cardiac follow-up recommendation.

## Discussion

Despite the significant reduction in mortality from CAD since 1980, it still accounted for 22% of all-cause mortality in women in 2013 [2]. The initial clinical presentation of CAD in women and men is often similar [3], but CAD is generally diagnosed in women who carry a greater burden of risk factors and who are approximately 10 years older than men [4]. Although the contribution of various risk factors to the development of acute coronary syndrome (ACS) has been extensively analyzed, women younger than 50 years old have rarely been included in clinical trials. Nevertheless, the effect of traditional risk factors have also been confirmed in this population: hypertension, smoking, and hyperlipidemia appear to have a high impact on the development of ACS in women [5], all of which were present in our patient.

At the same time, female gender could be associated with a higher 30-day mortality after an episode of ACS compared to males [6]. The reason for this disparity is still under debate. It appears that women more often have atypical ACS presentations and are less likely to undergo cardiac catheterization, timely reperfusion, or optimal medical therapy [7].

The pathophysiology of myocardial ischemia in women adds to the complexity of CAD management, as the female gender is associated with overlapping etiologies (non-obstructive CAD, microvascular or endothelial dysfunction, epicardial and microvascular spasm, myocardial bridging, and conduit vessel stiffening), all of which decrease the diagnostic accuracy of noninvasive tests [8]. Nuclear myocardial perfusion imaging is a noninvasive imaging modality that has been validated for use in symptomatic, intermediate-risk women, although it has some limitations caused by breast attenuation defects [9].

CCTA is emerging as a good diagnostic and prognostic imaging modality in patients suspected of CAD. Recent studies have shown that CCTA is as sensitive and specific in women as it is in men [8]. The positive and negative predictive values of CCTA have generally been identified as high [10], although the specificity is reduced by coronary calcium [11]. The prevalence of obstructive CAD as assessed by CCTA in low- to intermediate-risk patients was identified to range from as low as 18% [12-13] to as high as 32%-43% [14-15]. Min et al. suggested that CCTA findings successfully identify individuals at a higher risk of incident MACE [16]. Furthermore, the absence of plaques by CCTA portends a favorable prognosis for intermediate-term follow-up [11]. These findings have led to the use of CCTA in cases where invasive coronary angiography is generally used to rule out coronary artery lesions [17]. However, using clinical indexes further increases the ability of CCTA to predict MACE [18].

Ultimately, noninvasive testing portends limitations that must be weighed against the risks of invasive coronary angiography, which remains the gold standard for a CAD diagnosis [19]. Our case illustrates a situation where invasive testing for CAD was deferred based on reliable and consistent information from multiple non-invasive diagnostic modalities (nuclear stress testing, CCTA). However, an overall reluctance in clinical practice for performing invasive coronary procedures in women has been described in the literature [8]. This could be, in part, justified by the fact that from an epidemiological standpoint, women with chest pain are less likely than men to be diagnosed with CAD [20].

## Conclusions

A careful clinical history is seldom sufficient for the diagnosis of CAD as the etiology of chest pain. Stress testing is the traditional noninvasive approach to detecting obstructive CAD. An anatomic assessment of the coronary arteries should be considered in cases of nondiagnostic findings or a strong clinical suspicion conflicting with the findings on stress testing. CCTA has been validated as an imaging modality associated with the prediction of future MACE. Further studies are needed to improve the accuracy and the negative predictive value of CCTA in intermediate-risk individuals. Proposed markers of future plaque instability and coronary risk, such as the degree of vessel remodeling and low-attenuation plaque volume, as well as measures of CT myocardial perfusion, may additionally improve the prognostic value of CCTA.
